# Unveiling the 2017 *Karenia* Bloom in NW Chilean Patagonia by Integrating Remote Sensing and Field Data

**DOI:** 10.3390/microorganisms13112440

**Published:** 2025-10-24

**Authors:** Patricio A. Díaz, Raúl Gormaz, Paula Aguayo, Iván Pérez-Santos, Gonzalo S. Saldías, Rosa I. Figueroa, Pamela A. Fernández, Gonzalo Álvarez, Camilo Rodríguez-Villegas, Camila Schwerter, David Cassis, Rodrigo Vera, Carlos Conca

**Affiliations:** 1 Centro i~mar, Universidad de Los Lagos, Casilla 557, Puerto Montt 5480000, Chile; ivan.perez@ulagos.cl (I.P.-S.); pamela.fernandez@ulagos.cl (P.A.F.); camilo.rodriguez@ulagos.cl (C.R.-V.); camilaschwerter1@gmail.com (C.S.); 2 Centro de Biotecnología y Bioingeniería (CeBiB), Universidad de Los Lagos, Puerto Montt 5480000, Chile; 3 Departamento de Ingeniería Matemática, Universidad de Chile, Santiago 8330601, Chile; rgormaz@dim.uchile.cl (R.G.); cconca@dim.uchile.cl (C.C.); 4 Centro de Biotecnología y Bioingeniería (CeBiB), Universidad de Chile, Santiago 8320000, Chile; paguayo@dim.uchile.cl; 5 Centro de Investigación Oceanográfica COPAS COASTAL, Universidad de Concepción, Concepción 3349001, Chile; gsaldias@ubiobio.cl; 6 Centro de Investigaciones en Ecosistemas de la Patagonia (CIEP), Coyhaique 5950000, Chile; 7 Departamento de Física, Facultad de Ciencias, Universidad del Bío-Bío, Concepción 4051381, Chile; 8 Centro FONDAP de Investigación en Dinámica de Ecosistemas Marinos de Altas Latitudes (IDEAL), Valdivia 5090000, Chile; 9 Centro Oceanográfico de Vigo, Instituto Español de Oceanografía (IEO-CSIC), 36390 Vigo, Spain; rosa.figueroa@ieo.csic.es; 10 Departamento de Acuicultura, Facultad de Ciencias del Mar, Universidad Católica del Norte, Coquimbo 1780000, Chile; gmalvarez@ucn.cl; 11 Centro de Investigación y Desarrollo Tecnológico en Algas (CIDTA), Facultad de Ciencias del Mar, Universidad Católica del Norte, Larrondo 1281, Coquimbo 1780000, Chile; 12 Center for Ecology and Sustainable Management of Oceanic Islands (ESMOI), Departamento de Biología Marina, Facultad de Ciencias del Mar, Universidad Católica del Norte, Coquimbo 1780000, Chile; 13 AquaBC Chile SpA, Puerto Varas 5550505, Chile; davidcassis@gmail.com; 14 Departamento de Medio Ambiente, División de Acuicultura, Instituto de Fomento Pesquero, Balmaceda 252, Puerto Montt 5501248, Chile; rodrigo.vera@ifop.cl

**Keywords:** harmful algal blooms (HABs), *Karenia* spp., Sentinel-3, support vector classification (SVC), Chilean Patagonia

## Abstract

In southern Chile, harmful algal blooms (HABs) pose a threat to public health, artisanal fisheries, and the aquaculture industry (mussels and salmon). However, little is known about the environmental factors contributing to outbreaks of HABs in fjord systems. In summer 2017, an oceanographic cruise was carried out to study the physical processes associated with a bloom of the dinoflagellate *Karenia* spp. in the Gulf of Penas and Taitao Peninsula, Chilean Patagonia, causing a massive mortality of salmon (approximately 170,000 fish, worth USD 390,000). Satellite images from Sentinel-3 were utilized to distinguish between areas with high and low densities of *Karenia* cells. Cell densities were highest in the waters of the northern Taitao Peninsula (70 × 10^3^ cells L^−1^), and lowest at the Gulf of Penas. Support vector classification (SVC) based on bands 1 (400 nm), 2 (412.5 nm), and 6 (560 nm) from the Sentinel-3 images and the normalized fluorescence line height (FLH) classified bloom presence/absence with an 83% coincidence rate. The SVC model correctly identified non-bloom areas, with limited false positives, and successfully captured bloom zones where *Karenia* densities were highest. These results demonstrate the importance of incorporating satellite tools in the design and implementation of monitoring programs for the early detection of HABs, particularly in remote, difficult-to-access areas.

## 1. Introduction

Over the past few decades, increases in the frequency and intensity of harmful algal bloom (HAB) events, the expansion of their geographical range, and the consequent disruption of coastal activities have been reported [1]. However, in a recent meta-analysis of a global database of toxic/harmful events, the IOC-UNESCO HAEDAT (https://haedat.iode.org, accessed on 20 March 2025), the authors found no evidence of a clear global increase in HAB events over the past 30 years. Instead, they attributed the findings of previous studies to the exponential growth of monitoring observations [2]. In their recent State of the Ocean Report, the IOC-UNESCO highlighted the importance of assessing HAB dynamics at local and regional scales, including the impacts of multiple climatic and anthropogenic drivers and the species-specific ecological characteristics of the blooms [3]. The latter have become particularly relevant given the emergence of new HAB-forming species and their detection in previously unaffected areas [4,5]. Severe HABs in recent decades have caused massive mortalities of native and farmed marine fauna [6,7], prolonged shellfish harvesting bans due to biotoxin levels exceeding regulatory limits [8,9,10], and outbreaks of human poisoning, including fatal cases [11]. These consequences have highlighted the need for a better understanding of the complex factors that drive and modulate HAB events.

In Chilean Patagonia, the recurrence of ichthyotoxic HABs of species such as *Pseudochattonella verruculosa* [12,13], *Heterosigma akashiwo* [6,14], and *Karenia* spp. [15,16,17,18] threaten fisheries and aquaculture [6,14], including salmon aquaculture in Patagonian fjords [11,19,20]. Nonetheless, the focus of the few studies on ichthyotoxic microalgae [13,19] has mostly been on the microalgal species responsible for paralytic shellfish poisoning (PSP) [21,22], diarrheic shellfish poisoning (DSP) [23,24,25], and amnesic shellfish poisoning (ASP) [11,26]. In addition to the limited knowledge of the physiology, life cycle, and bloom dynamics of ichthyotoxic species in general, the complex, remote geography of Patagonian fjords and channels [27] hinders regional studies of bloom formation and evolution. Consequently, species of the genus *Karenia*, an unarmored marine dinoflagellate and emerging genus in Patagonian fjords [15,17], have been poorly studied. Although *Karenia* species are found in oceanic and coastal waters, they were considered to be present in warmer, and more saline waters that support their ecological preferences [28]. For example, the optimal growth of *Karenia brevis* occurs at salinities between 30 and 34 [29,30], and that of *Karenia selliformis* at a salinity of 30 and a temperature of 18 °C [18]. These characteristics have contributed to the recent emergence of both species in the outermost waters of Patagonian fjords, but also *K. brevis*, *K. papilionacea*, *K. mikimotoi*, *K. brevisulcata*, and *K. bidigitata*, have been observed in this area only in high-salinity (>34) waters [15]. This worrisome trend of emergent species can be worse if considered that some *Karenia* spp., develop sexual resting cysts as observed in *K. mikimotoi* in sediments from the East China Sea [31], adding to their emergence a spatio-temporal persistence through the shift in their life cycle, coupling with a planktonic–benthonic habitat.

In this sense, owing to some HABs outbreaks might occur in remote/difficult to access areas, the use of satellite images to monitor HABs rise up an effective alternative that has advanced in recent years. For example, environmental variables such as sea surface temperature (SST) and chlorophyll-*a* (Chl-*a*) have been analyzed using the Sea-viewing Wide Field-of-view Sensor (SeaWiFS), MEdium Resolution Imaging Spectrometer (MERIS), and Moderate Resolution Imaging Spectroradiometer (MODIS) instruments [32,33,34]. The MERIS mission operated with 15 bands (412.5–900 nm) and a full spatial resolution of 300 m [35], while MODIS employed nine ocean color bands (405–877 nm) and a full spatial resolution of 1000, 500, or 250 m, depending on the band (National Aeronautics and Space Administration). A recent mission, Sentinel-3 Ocean and Land Colour Instrument (OLCI), was designed based on ENVISAT’s MERIS mission and has shown excellent results for marine applications. Sentinel-3 effectively combines a high spectral resolution (21 bands, 400–1020 nm) with a spatial resolution of 300 m [36]. This spatial resolution should be enough to monitor some HAB events, considering that it is well accepted that phytoplankton populations can be geographically structured in open ocean environments, which means that dispersal and migration patterns or species populations boundaries can be revealed in areas as small as 100 km and in time frames of months ([37] and references there in). So given its advanced capabilities, Sentinel-3 has already been successfully applied to monitor HAB events [17,38,39,40,41,42].

**Figure 1:**
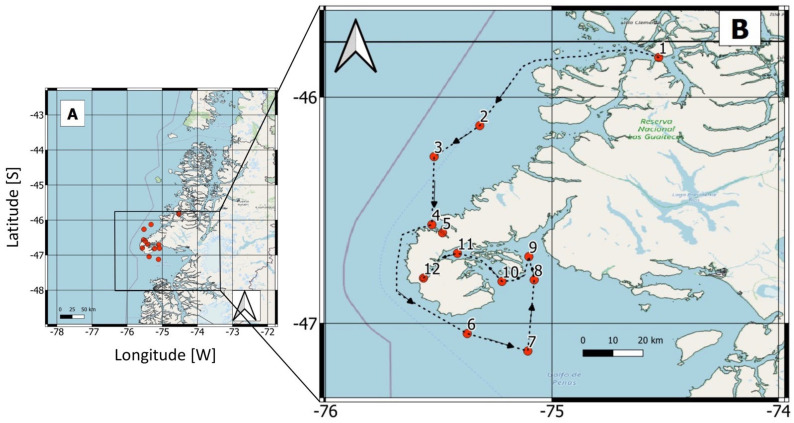
Map of the study area, showing (**A**) Northwest Patagonia. The boxed area encloses the central/northern part of the Gulf of Penas, southern Taitao Peninsula, and the 12 oceanographic sampling stations (red circles). (**B**) Close-up of the locations of the 12 sampling stations (red circles) visited during the February 2017 oceanographic cruise.

A thorough understanding of the short-term variability in HABs is essential for the development of reliable operational models [43,44] and to improve risk assessments of shellfish poisoning and other hazardous events. In the present study, Sentinel-3 satellite data were combined with in situ data to determine the environmental conditions that promote the development of dense *Karenia* blooms in the Gulf of Penas, Chilean Patagonia (Figure 1). Thus, the new field-satellite matchups for *Karenia* bloom in a remote area in Chilean Patagonia, the Gulf of Penas, advance our understanding of these types of toxic ocean blooms, not previously addressed with this methodological approach in systems as complex as the fjords and channels of Chilean Patagonia.

## 2. Materials and Methods

### 2.1. Study Area

Chilean Patagonia is one of the most extensive fjord regions in the world (Figure 1), with ~240,000 km^2^ of islands, peninsulas, fjords, and channels [27]. In addition to the region’s abrupt bathymetry and complex coastal morphology, its waters are highly stratified, due to intense freshwater runoff and elevated precipitation [45]. Furthermore, its geographical configuration limits the free exchange of water between coastal areas and the open ocean, which promotes the formation of microhabitats supporting a highly productive ecosystem [46].

The Gulf of Penas (47° S), located in the southern Aysen Region, forms part of the extensive Patagonian fjord system. It extends inland for 90 km and covers a stretch of approximately 80 km southwards, from the Taitao Peninsula to the Guayaneco Archipelago. Oceanic waters in the area have a salinity of ~32 at the surface [47]. The surface salinity distribution displayed a zonal gradient in which lower salinities (<33) were observed inside the fjords/channels and higher salinities (33–34) are registered in the adjacent open ocean owing to the presence of the Subantarctic Water (SAAW) [48]. South of 46° S, the region is influenced by downwelling winds that force the onshore transport of surface water [49,50].

### 2.2. Field Sampling

From 21 to 26 February 2017, a research cruise took place in southern Chile on board the PSG “Contramaestre Ortiz”, belonging to the Chilean Navy. During the cruise, 12 sampling stations (Figure 1B) were visited, where vertical profiles of temperature, salinity, and in vivo fluorescence were obtained using a self-contained autonomous micro-profiler (SCAMP) system. The SCAMP profiler records data at 100 Hz with a descending free-fall speed of ~10 cm s^−1^. Data were also collected from a conductivity, temperature, and depth (CTD) profiler equipped with a dissolved oxygen (DO) sensor. CTD profiles were graphically represented using Ocean Data View [51] and the DIVA algorithm to interpolate between sampling stations.

During the cruise, seawater samples (125 mL) for quantitative analyses of phytoplankton were collected at three depths [surface, ~5 m (maximum chlorophyll), and 25 m] using Niskin bottles (5 L each). Additionally, plankton nets (20-μm mesh) were hauled vertically (0–20 m) to collect phytoplankton for qualitative analyses. All phytoplankton samples were immediately fixed with an acidic Lugol’s solution [52].

### 2.3. Phytoplankton Analysis

For quantitative analyses of phytoplankton, 10 mL of unconcentrated acidic Lugol’s-fixed sample was left to sediment for 24 h and then analyzed under an inverted microscope (Olympus CKX41, Olympus, Tokyo, Japan) using the method described in Utermöhl [53]. To enumerate large species, the entire surface of the chamber was scanned at a magnification of ×100 to ensure a detection limit of 100 cells L^−1^.

### 2.4. Satellite Data

High-resolution (300 m) Sentinel-3 images were obtained for the period between 20 February and 1 March 2017, to coincide as closely as possible with the field sampling dates. The sensor has a field of view of 68.5° at nadir and covers a swath width of 1270 km at an altitude of 814.5 km, allowing for a revisit time of <2 days. Ocean-color studies are possible using Sentinel-3 OLCI’s 21 bands. The spatial resolution on the ground is 300 m. The spectral band characteristics are described in Appendix A.

Sentinel-3 OLCI level-1 data were obtained from the Copernicus open access hub (https://browser.dataspace.copernicus.eu/) on 4 March 2021. The images are projected in the system under the reference EPSG:32718–WGS 84/UTM zone 18S and radiometrically calibrated by the European Space Agency (ESA) using on-board calibration systems. Therefore, no additional radiometric adjustment was required. Subsequent preprocessing included subsetting to the study area, cloud masking, and visual inspection to verify geometric alignment between scenes and correspondence with in situ sampling points. These steps ensured that the radiometric and geometric quality of the imagery met the requirements for computing the spectral variables used in the analysis.

Image correction for atmospheric effects was performed with the iCOR software [54], which uses the MODerate resolution atmospheric TRANsmission (MODTRAN5) code [55] and is publicly available from the Flemish Institute for Technological Research (VITO). Thus, the iCOR-OLCI plugin for SNAP toolbox, version 3.0, was used.

Following the atmospheric corrections, the normalized water-leaving radiance (nLw(λ), mW cm^−2^ μm^−1^ sr^−1^) was calculated by multiplying the remote sensing reflectance (Rrs) by the mean solar irradiances (Fo); pixels corresponding to land or cloud-covered areas were discarded.

Standard procedures were used to estimate the following: (1) Chl-*a* concentration [56]; (2) the Normalized Difference Chlorophyll Index (NDCI) [57]; (3) fluorescence line height (FLH) [58]; and (4) the diffuse attenuation coefficient (Kd). Some of these algorithms were designed for MODIS images, whose spectral characteristics slightly differ from those of Sentinel-3. These differences and the algorithm adaptation used to account for them are shown in Appendix A and Appendix B. Twenty predictive variables were used, 16 of which corresponded to bands related to atmospheric correction and 4 to derived products.

### 2.5. Identification of Karenia Bloom from Satellite Images

Soto et al. [33] reviewed several algorithms designed to detect blooms of *Karenia brevis*. Although this species has not been identified in Chilean waters, these algorithms can be adapted for species of the genus *Karenia*. Chl-*a* images, FLH, and Rrs(λ) were also used in the assessments. The Red Band Difference (RBD) algorithm could not be applied, due to its poor performance, as these authors only obtained a correct prediction 7 out of 13 cases.

Instead, the RBD value was computed using bands 13 and 14 from MODIS, as shown in Equation (1):

(1) $$RBD=nLw\left(678\right)-nLw(667)$$

This can be approximated from bands 8, 9, and 10 of Sentinel-3 as shown in Equation (2):

(2) $$RBD=nLw\left(673.75\right)+\frac{nLw\left(681.25\right)}{2}-nLw(665)$$

In a more elaborate strategy, satellite image data obtained from sites showing the presence of blooms can be compared with data from images of sites in which blooms were absent. A bloom was defined as a cell density > 800 cells L^−1^. Ideally, this strategy would be deployed at the same geographic location but at different times. Various statistical methods can be used (primarily regression and classification methods) to identify areas marked by the presence of blooms and distinguish them from areas in which blooms are absent. This method of data analysis can then be adapted to enable a machine-learning-based approach to the analysis of new data and thus to the detection of new blooms. A prerequisite for this type of generalization and, in this case, the recognition of new blooms, is a sufficient variety of data and data vectors.

Although our study did not fully meet the conditions needed for robust predictive modeling, its results provide a valuable conceptual framework and highlight the potential of machine learning methods as a preliminary step in an in-depth analysis. Specifically, we examined 12 records associated with the *Karenia* blooms that occurred in the Gulf of Penas during the summer of 2017. Each record included *Karenia* cell densities (cells L^−1^) and a vector of 23 data items. Regression and classification methods were applied and showed the low power of generalization, as expected. Support vector classification (SVC) was performed using the LIBSVM, a library for support vector machines. The use of this computational package was limited to a vector consisting of four parameters, as described in Chang and Lin [59], which reduced the risk of overfitting (too many parameters but too few data). The SVC was trained using spectral variables derived from Sentinel-3 OLCI imagery, including individual reflectance bands and the FLH index. Prior to training, all input variables were standardized using the StandardScaler function to ensure unit variance. The four parameters were selected using the Recursive Feature Elimination (RFE) method. The algorithm was based on Equation (3):

(3) $$V=0.9091256\times FLH-0.39982808\times band_{1}+0.56550125\times band_{2}-0.6003501\times band_{6}-3.6486782$$

The calculation was based on bands 1 (400 nm), 2 (412.5 nm), and 6 (560 nm) from the Sentinel-3 OLCI images and the normalized FLH. The selected Sentinel-3 OLCI bands were chosen for their sensitivity to chlorophyll-a absorption and cellular backscattering typical of phytoplankton blooms [60,61,62].

For classification purposes, if the computed *V* value for a given pixel was greater than zero, the pixel was labeled as a bloom, whereas all other values (≤0) indicated non-bloom conditions. This threshold directly corresponds to the decision boundary of the trained linear SVC model.

### 2.6. Statistical Analysis

The effects of the physicochemical conditions of the water column (temperature, salinity, oxygen, and fluorescence) on the phytoplankton assemblages obtained during the oceanographic cruise were compared in a constrained analysis of proximities (CAP) in the Jaccard distance matrix, performed using the “capscale” function in the “vegan” package from R [63,64]. CAP is an exploratory ordination method that is useful in visualizing the effect of selected variables over the entire phytoplankton assemblage.

The hypothesis test was evaluated based on the water column conditions and the species composition of the phytoplankton assemblages. A marginal permutational analysis of variance (PERMANOVA) based on Jaccard dissimilarities [65,66] was applied using the “adonis2” function from the R package “vegan” [67]. An empirical pseudo-*F* distribution and the *p*-values were calculated from 10,000 permutations. The robustness of the PERMANOVA hypothesis test was demonstrated using severely non-normal and zero-inflated ecological/field data [65]. The statistical resolution thus obtained derived from the identification of the contribution of specific variables to the phytoplankton community.

Following the recommendations of the American Statistical Association [68] and an increasing number of scientists worldwide [69], the *p*-values were not dichotomized, but are instead reported as calculated, except when several values were combined to represent a single group.

## 3. Results

### 3.1. Hydrographic Characterization

**Figure 2:**
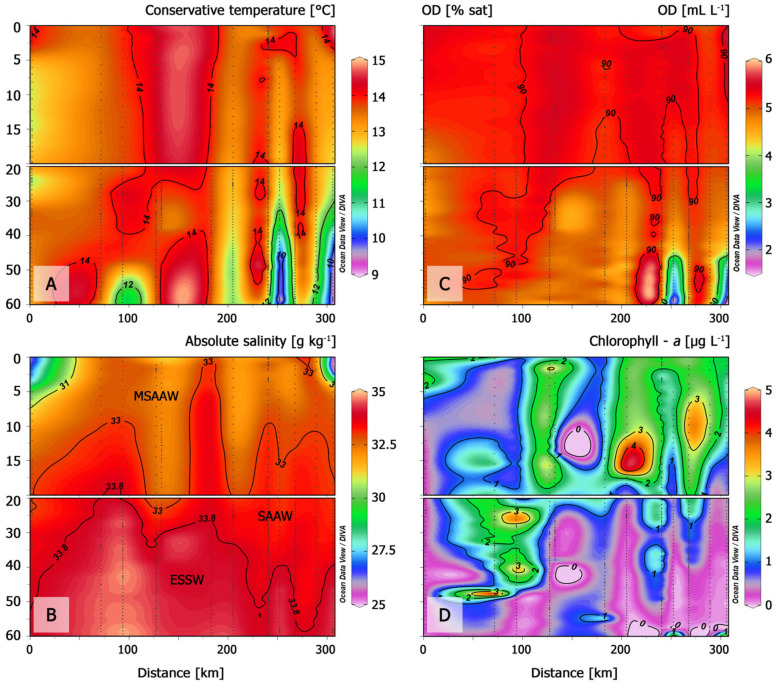
Vertical distribution of: (**A**) conservative temperature (°C), (**B**) absolute salinity (g kg^−1^), (**C**) dissolved oxygen (mL L^−1^), and (**D**) chlorophyll-*a* (µg L^−1^) at 12 sampling stations located within a 320 km transect, as measured during a February 2017 oceanographic cruise. Dotted vertical lines correspond to CTD profiles. Interpolation between sampling stations was performed using the DIVA algorithm from Ocean Data View.

Hydrographic data were collected in a transect that began in Darwin Channel (station 1), and continued in the open ocean (stations 2–4), concluding in the Gulf of Penas (stations 6–10) and Slight Bay (stations 11 and 12) (Figure 2).

Surface temperatures ranged from ~11 °C within the fjord and channels to ~15 °C at the Gulf of Penas (Figure 2A). A clear thermocline characterized the upper 20–30 m, with cooler water masses (~9–10 °C) appearing at depths below 40 m. The coldest subsurface water corresponded to the presence of equatorial subsurface water (Figure 2B). A well-mixed layer in terms of temperature and salinity, denoting a subantarctic water mass, was present between these stations. Estuarine and modified subantarctic waters were detected in the upper 20 m of the water column at the edges of the hydrographic transect. 

In general, mixing conditions dominated, although strong stratification, with a sharp halocline, occurred at ~5 m depth at stations 1 and 12 (Figure 2B). DO concentrations were high at stations located in the open waters of the Pacific Ocean and Gulf of Penas, with values < 6 mL L^−1^ and 90% saturation extending from the surface layer to −30 m (Figure 2C). These areas of high DO concentration coincided with those of high fluorescence (Figure 2D). Maximum fluorescence levels at stations 2 and 3 were detected not only close to the surface layer but also at ~50 m depth.

### 3.2. Distribution of Karenia Cells

**Figure 3:**
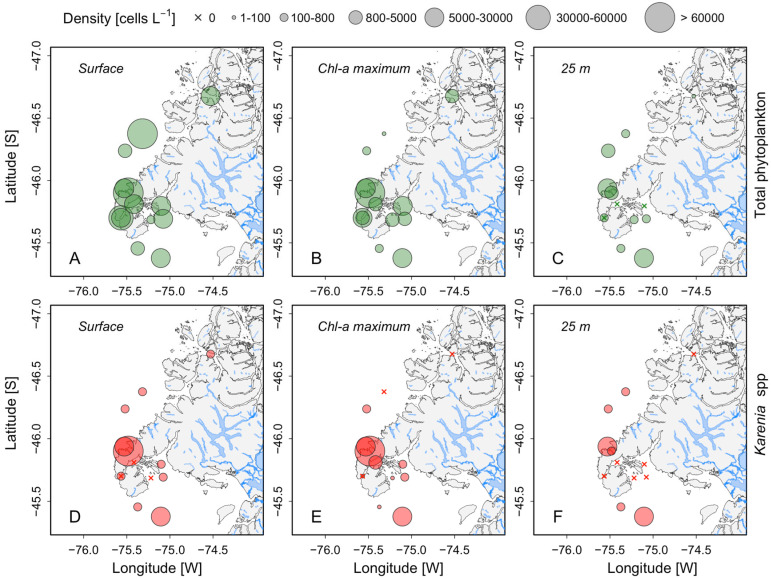
Spatial distribution of total phytoplankton (upper panels) and *Karenia* spp. (lower panels) (cells L^−1^) as recorded during a February 2017 oceanographic cruise. Water samples were obtained from: (**A**,**D**) the surface, (**B**,**E**) at the chlorophyll-*a* maximum, and (**C**,**F**) 25 m depth.

In February 2017, a multi-specific bloom of *Karenia* spp. (mainly *Karenia* cf. *mikimotoi*, *K. brevisulcata* and *K. papilionacea*) was observed in the Gulf of Penas (Figure 1). The phytoplankton analysis revealed a community dominated by dinoflagellates, primarily *Kareniaceae*, which accounted for 54–99% of the total phytoplanktonic community at stations where the density of *Karenia* spp. exceeded 5000 cells L^−1^ (Figure 3). At stations where *Karenia* was not detected, the phytoplankton community was dominated by diatoms, mainly *Leptocylindrus danicus* and *Paralia sulcata*.

**Figure 4:**
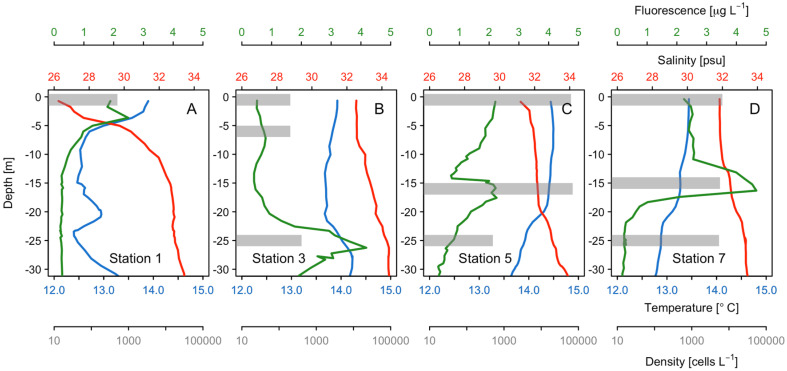
Vertical distribution (0–30 m) of temperature (blue line), salinity (red line), chlorophyll-*a* fluorescence (green line), and *Karenia* spp. density (cells L^−1^, gray bar) at sampling stations 1 (**A**), 3 (**B**), 5 (**C**), and 7 (**D**).

Off the northern side of Taitao Peninsula, maximum cell densities of *Karenia* spp. were approximately 62.8 × 10^3^ cells L^−1^ at the surface and 70.4 × 10^3^ cells L^−1^ at 5 m (Chl-*a* maximum) depth (station 5; Figure 3 and Figure 4), accounting for 98.9% and 94% of the total phytoplankton community, respectively (Figure 3D,E).

At station 7, *Karenia* spp. densities reached a maximum of 5.7 × 10^3^ cells L^−1^, accounting for 54% of the total phytoplankton community and coinciding with the Chl-*a* maximum at 15 m, but the bloom was less intense than the one at station 5 (Figure 4). The density of *Karenia* spp. was higher at surface layers with warmer (>14 °C) than colder (~12.5 °C) waters (Figure 4).

### 3.3. Satellite Bloom Detection

**Figure 5:**
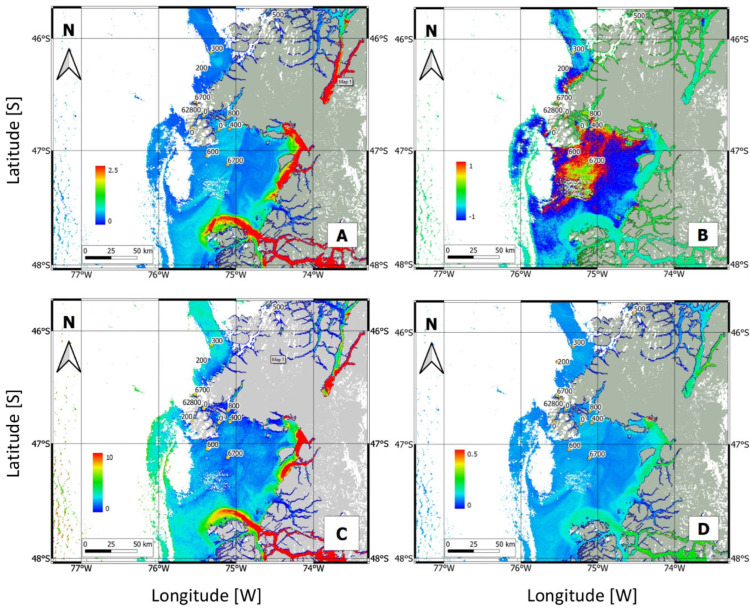
Maps of the study area in Northwest Patagonia showing: (**A**) the Chl-*a* concentration; (**B**) the normalized difference chlorophyll index (NDCI); (**C**) the fluorescence line height (FLH); and (**D**) the diffuse attenuation coefficient (Kd). In all four maps, the blue color represents minimum values of the index, while the red color represents maximum values of each. Masking cloud is denoted by white color and masking land by gray color.

The Chl-*a* concentration can act as a warning of an algal bloom. The concentrations measured in the study area are shown in Figure 5A. River plume areas with high turbidity are colored red, and the central Gulf of Penas (CGP) and northern Taitao Peninsula (NTP) in light blue. The NDCI, a qualitative measure of the Chl-*a* concentration and algal blooms, is shown in Figure 5B, where the CGP and NTP are colored red.

The FLH is an additional early warning signal of algal blooms but, unlike the Chl-*a* concentration, it is not biased by highly turbid waters. Figure 5C presents the FLH results for the CGP and NTP (shown in light blue). The Kd for the downward plane irradiance at 490 nm is a seawater transparency product and is shown in Figure 5D, where turbid river plume areas are colored green, and the CGP and NTP light blue. The turbid river plumes, representing freshwater delivered into the Gulf of Penas, had high FLH and Chl-*a* values, but not high NDCI values.

Table 1 summarizes the agreement between the observed *Karenia* bloom events (cell density ≥ 800 cells L^−1^) and the events predicted by the model using the computed RBD algorithm across the 12 sampling stations. For each station, the table reports whether a bloom was observed in situ, the RBD value computed using the model, the binary prediction (bloom or no bloom), and whether the prediction coincided with the observation.

**Figure 6:**
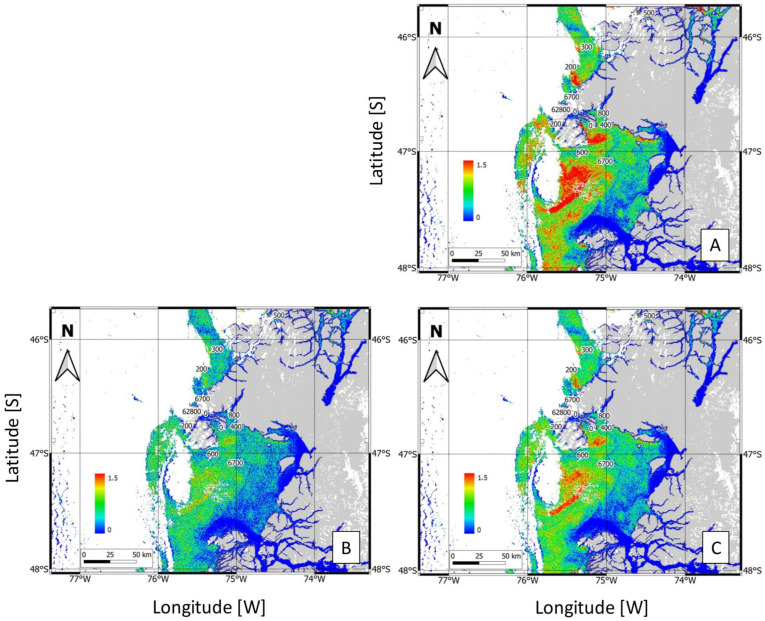
Maps of the study area in Northwest Patagonia showing the results from the RBD algorithm: (**A**) the difference between band 10 (nLw 681.25) and band 8 (nLw 665); (**B**) the difference between band 9 (nLw 673.75) and band 8 (nLw 665); and (**C**) the difference between the average of bands 10 and 9 and band 8. In all three maps, the blue color represents minimum values of the index, while the red color represents maximum values of each. Masking cloud is denoted by white color and masking land by gray color.

From the 12 stations included in this study, the model correctly predicted the presence/absence of blooms in 6 of them, resulting in a 50% coincidence with the observations based on the field samplings. False positives, in which blooms were predicted but not observed, occurred at stations 1, 2, 3, 8, and 10, whereas a false negative, in which a bloom was observed but not predicted, occurred at station 5. The correct matches (coincidence = 1) at stations 4, 6, 7, 9, 11, and 12 represent instances in which the model aligned with actual observations. These results suggest that, despite the potential of the RBD-based model for predicting bloom events, further refinement is needed to reduce the number of incorrect predictions and improve reliability. Compared with the MODIS image bands used by the RBD algorithm, the closest Sentinel-3 bands are band 8 (665 nm), band 9 (673.75 nm), and band 10 (681.25 nm). Figure 6 shows the spatial output of the RBD algorithm using the three different spectral band combinations to detect surface *Karenia* blooms in the Gulf of Penas and adjacent fjords.

The RBD was computed as the difference between band 10 (nLw 681.25 nm) and band B8 (nLw 665 nm). The areas of intense bloom activity (red to yellow areas in Figure 6) were concentrated primarily in the central region of the Gulf of Penas and Northern Taitao Peninsula (Figure 6A). When the RBD was calculated based on the difference between band 10 (nLw 673.75 nm) and band 8 (nLw 665 nm), the spatial distribution of bloom-prone areas was broader and bloom intensity was lower, with diminished signal intensity in some of the previously highlighted zones (Figure 6B). An intermediate pattern, with a more continuous spatial coverage by elevated RBD values across the bloom region, was obtained when the RBD was calculated as the difference between the average of bands 10 and 9 and band 8 (Figure 6C).

A comparison between the observed bloom events and the predictions derived from the *V* equation across the 12 monitoring stations is presented in Table 2. For each station, the observed bloom condition (1 = bloom, 0 = no bloom), the computed *V* value, the binary prediction based on that value, and whether or not the prediction matched the field observations are reported.

Among the 12 stations, the model correctly classified bloom presence/absence at 10 stations, resulting in an 83% coincidence rate. Most of the correct predictions corresponded to stations where no bloom was observed (stations 1, 2, 3, 6, 8, 10, 11, 12). The model also correctly identified the blooms at stations 4 and 7. However, it failed to predict the bloom events at stations 5 and 9. Thus, while the *V*-based model successfully identified non-bloom conditions, it was limited in detecting bloom presence (false negatives), suggesting a conservative bias in its predictive behavior.

**Figure 7:**
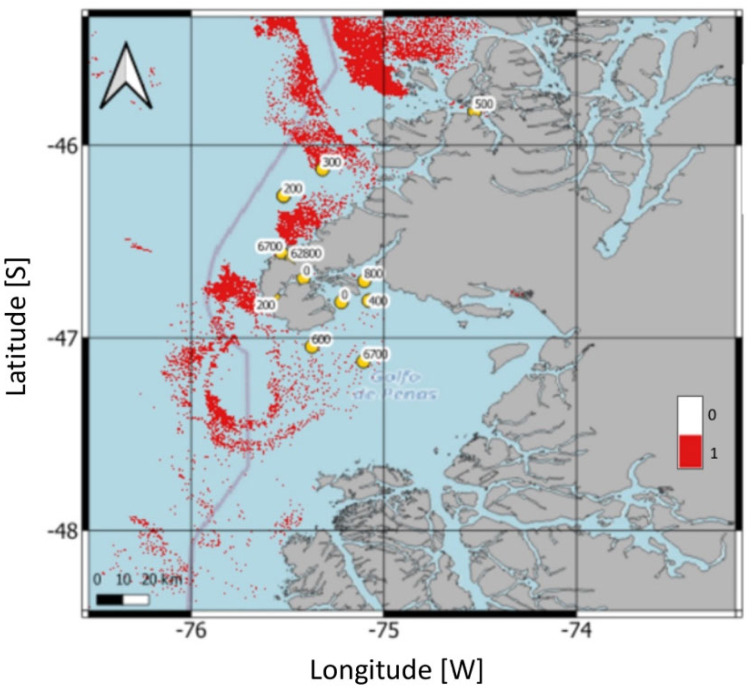
Maps of the study area in Northwest Patagonia showing the results of the support vector classification. Red pixels (*V* > 0) indicate areas of *Karenia* blooms. The maximum detected cell densities (cells L^−1^) of *Karenia* are shown as yellow dots at each sampling station. Masking land is denoted by gray color.

The spatial distribution of the predicted *Karenia* bloom areas based on the SVC model is shown in Figure 7 and corresponds to the outcomes of the *V* equation reported in Table 2. Red pixels represent areas of *V* > 0, indicating bloom-positive predictions. Yellow dots show the locations of field sampling stations, annotated with the maximum observed cell densities (cells L^−1^) of *Karenia*.

The figure confirms a strong spatial match between predicted bloom areas and known bloom hotspots in the Gulf of Penas, especially along the southwestern coast of Taitao Peninsula (Figure 2). This visual output consistent with the high prediction accuracy (Table 2), in which 10 out of 12 stations were correctly classified. Moreover, the SVC model was able to correctly identify non-bloom areas, with limited false positives, and successfully captured bloom zones near sampling stations where *Karenia* densities were highest. The two false negatives, at stations 5 and 9, were not located within dense red pixel clusters, reflecting the model’s conservative approach and limited sensitivity in some bloom-positive areas.

### 3.4. Statistical Analysis

**Figure 8:**
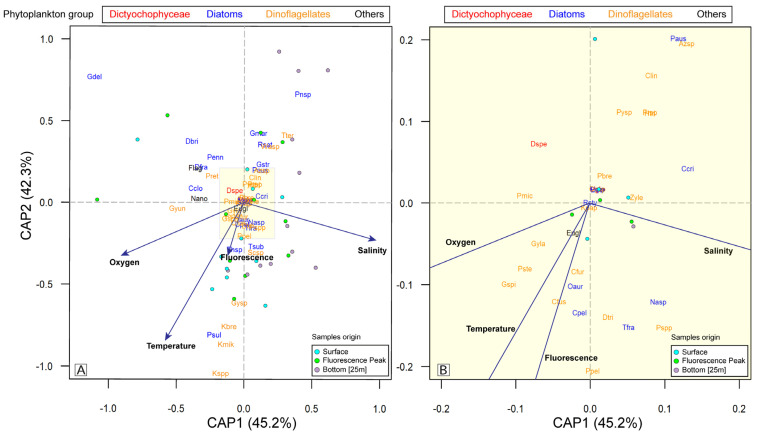
Ordination tri-plot of a constrained analysis of principal coordinates (CAP) in the Jaccard distance matrix, evaluating the effects of environmental variables on the phytoplankton assemblages determined in water samples from the oceanographic cruise conducted in the austral summer of 2017. (**A**) CAP ordination with all species with a zoom insert (yellow area). (**B**) Amplification CAP ordination of the yellow area insert. A key to the species names is provided in Appendix C.1.

The CAP ordination showed marked differences in the species composition of the phytoplankton assemblages collected from the surface, the depth of maximum fluorescence, and the bottom (~25 m) (Figure 8). Cumulatively, the first two axes represented 87.5% of the variation in the Jaccard distance matrix of the assemblages (Figure 8). In the CAP analysis, two environments could be distinguished: one characterized by the correlation of temperature, fluorescence, and oxygen (bottom left), and the other by salinity (bottom right) (Figure 8).

The abundances of toxigenic species, such as *K*. *brevis*, *K. mikomotoi*, and *Karenia* spp., were strongly influenced by temperature, oxygen, and fluorescence but not by salinity (Figure 8). This pattern was also observed for the diatom *Paralia sulcata* and the dinoflagellate *Gyrodinium* spp. (Figure 8). By contrast, according to the results of the PERMANOVA, salinity (*p* = 0.049), temperature (*p* = 0.056), and oxygen (*p* = 0.069) strongly influenced the species composition of the phytoplankton community, whereas the effects of the fluorescence predictor variable were not significant (Table 3). These results were consistent with those obtained in the CAP analysis.

## 4. Discussion

In recent decades, the occurrence and intensity of HABs have increased in southern Chile due to environmental conditions that have altered the oceanographic dynamics of Patagonian fjords and channels. For instance, a decreasing trend in precipitation and in river discharge into coastal waters with site-specific patterns in water stratification has been observed [70]. Furthermore, upwelling-favourable winds offshore are also predicted for Northern Patagonia [71], which can be merged with the increasing trend in sea surface temperature as a result of higher values of solar radiation and with the more recurrent rate of heat waves [72]. These shifts might open a window of opportunity for population growth [73] that can promote the proliferation of ichthyotoxic microalgal species, such as *Pseudochattonella verruculosa*, *Heterosigma akashiwo*, and *Karenia* spp., (among other species), and have significantly impacted the local salmon industry several times, including mass mortalities of fish. The blooms in 2016 were the largest fish-killing events on record in the region [13,19] and were followed in the summer of 2017 by another large-scale salmon mortality event, resulting from a *Karenia* bloom in the Gulf of Penas [15]. To better predict algal bloom occurrences and thereby prevent their potentially adverse consequences, this study integrated in situ measurements (related to the physical properties of the water column) with satellite observations (Sentinel-3) to characterize the oceanographic conditions associated with *Karenia* spp., bloom. This approach was also applied to map the spatial extent of the 2017 bloom.

We are aware that the use and application of advanced technologies like this in HAB detection, prevention, and mitigation is far from its accurate implementation, but this approach can be joined to machine learning techniques that might help identify *Karenia* HABs in their initial phase so that management protective measures and appropriate steps can be taken by the local/regional stewardship to reduce bloom impacts. All of these aspects need to be operationally implemented under a precautionary principle approach.

### 4.1. Satellite Tools and Classification Method for Bloom Detection

The cell density of the *Karenia* spp., bloom recorded in the Gulf of Penas in mid-summer 2017 was lower (maximum 70 × 10^3^ cells L^−1^) than reported for the frequent *K. brevis* blooms in the Gulf of Mexico (>10^6^ cells L^−1^) [33,74], or the huge bloom of 2023 in southwest Florida (~388 × 10^6^ cells L^−1^) reviewed by Oh et al. [75]. However, the bloom reported here was very similar to the cell densities of the *Karenia* events in the fjord system of northwestern Patagonia in the late summer of 2020, with ~1.4 × 10^5^ cells L^−1^ [17]. Interestingly, the identified signal of total phytoplankton was most explained by the presence of *Karenia* spp. (see Figure 3). This pattern matches with some examples obtained from the Harmful Algae Event database (haedat) in which co-occurring blooms of *Karenia* spp., with species like *Heterosigma akashiwo*, showed less intensity (i.e., in terms of cell density) than in monospecific bloom at least for co-occurring blooms in Liadong Bay of Liaoning Province (China), Wakayama, and Yatsushiro Sea (Japan) [75], and Penas Gulf (Chile) in this study. However, in southern Chile, *Karenia* (“ex-*Gymnodinium* spp.) blooms with high cell density have also been recorded in Quellon Bay, where high mortality in shellfish (clam, sea urchins, abalone), farmed salmon, and wild fish species were affected, reaching ~8 to 9 × 10^6^ cells L^−1^. Moreover, the 2017 bloom was highly toxic and, as noted above, caused the massive mortality of salmon (approximately 170,000 fish, worth USD 390,000). At the time of their interaction with *Karenia* spp., and death, the fish were being transported on wellboats through the Gulf of Penas, where the bloom was in progress [15,16]. The boats extract water from surface layers (0–10 m), where cell densities are highest, resulting in a high toxicity for exposed fish.

High-resolution satellite sensors can be employed to identify a bloom’s surface distribution, which facilitates its monitoring [33,40,76]. This was demonstrated by Rodríguez-Benito et al. [40], who used the same satellite imagery (Sentinel-3) to track the temporal evolution and distribution of a bloom by the ichthyotoxic dinoflagellate *Cochlodinium* sp. and a high-biomass HAB by the dinoflagellate *Lepidodinium chlorophorum* in northwest Chilean Patagonian fjords during the late summer of 2020. Both events were characterized by high cell densities (*L. chlorophorum*: >5 × 10^6^ cells L^−1^) and intense changes in water color (Chl-*a* maximum of 20 μg L^−1^). By contrast, the 2017 *Karenia* bloom in the Gulf of Penas did not have high cell densities and therefore did not cause obvious color changes in the water column (Chl-*a* maximum of 2.5 μg L^−1^). However, field observations showed that the highest cell densities of *Karenia* occurred at sampling stations where the environmental conditions supported bloom formation—with temperatures ranging from 13.99 to 14.44 °C, salinity values of 31.2–32.71, and an oxygen saturation of 93.17–94.29%—and the fluorescence values were high, ranging from 2.21 to 3.56 µg L^−1^. Thus, despite the utility of satellite tools, field data are essential for validating their findings.

In this sense, it is important to clarify that nutrient analysis during the oceanographic campaign could be useful to know how much of the vegetative growth observed in situ was supported by these sources. Notwithstanding, *Karenia* species display high adaptability to nutritional sources and light preferences, being a mixoplanktonic strategist [17]. Mitra et al. (2016), refer to this dinoflagellates as photo/osmo/phagotrophs which means that their vegetative growth can be supported by photosynthesis even at low-light environments (with 10–20 plastids in their cytoplasm, Li et al. [77]), can take up dissolved organic matter by osmosis and phagocyte small cyanobacteria present in the environment [78]. *Karenia* spp., also succeed on regenerated N sources, such as ammonia, urea, and polyamines from animal waste or from diatom blooms in decomposition [17]. At this point, even when nutrient data are lacking, this species can grow and persist in different kinds of environments due to its great adaptability. On the other hand, although the Chl-*a* concentration is a useful indicator of the presence and evolution of a microalgal bloom, satellite-based determinations of HAB events are not always accurate, especially in areas such as Chilean Patagonia, where the many fjords and channels result in optically complex waters. Lara et al. [79] compared a large dataset of in situ Chl-*a* measurements collected between 2003 and 2012 with the MODIS images obtained as part of the CIMAR-FIORDOS program [80]. They found a low correlation (*R*^2^ = 0.2) between in situ and satellite-determined Chl-*a* levels. In general, satellite measurements may overestimate Chl-*a* concentrations due to interference by other compounds, such as colored dissolved organic matter, and shallow sea beds [81]. Our results showed low concentrations in the CGP and NTP (light blue in Figure 5A) and high concentrations in coastal waters close to river discharge areas (red in Figure 5A), where inputs of suspended sediments and terrestrial materials are high. Because the optical properties of those waters are usually influenced by sediment resuspension, organic matter, and mineral particles, their high Chl-*a* levels do not necessarily indicate an algal bloom.

The NDCI was initially developed to predict Chl-*a* concentrations in estuarine and coastal productive waters [57], but it is also useful for detecting algal blooms and for qualitative inferences of Chl-*a* concentrations in remote coastal waters with no ground truth data. In this study, the NDCI indicated high Chl-*a* concentrations (red pixels) in areas (central region of the Gulf of Penas and Northern Taitao Peninsula) where maximum *Karenia* cell densities were detected. It should be noted that the NDCI does not record the high Chl-*a* concentrations of turbid river plumes, in contrast to the Chl-*a* concentration algorithm (Figure 5). The latter was used by Rodríguez-Benito et al. [40] for the detection and monitoring of harmful dinoflagellate blooms in the Inner Sea of Chiloé (−43.5° S), Chile, in the late summer of 2020.

The FLH is a relative measure of the amount of radiance emitted from the sea surface, which is presumably due to chlorophyll fluorescence. Both the FLH and the Chl-*a* concentration algorithm were able to differentiate between the presence and absence of a *Karenia* bloom (light-blue pixels in Figure 5C). However, the FLH also detects fluorescence in areas of river discharge, which might be associated with local phytoplankton fluorescence. The Kd (Figure 5D) similarly highlights river drainage areas. These results explain why, in Figure 5C,D, the central region of the Gulf of Penas and Northern Taitao Peninsula are shown in light blue, indicating the possible presence of *Karenia*.

### 4.2. Implications for Monitoring Programs and Potential Impacts

The optically complex waters of Patagonian fjords and channels limit the use of satellite images alone in detecting and monitoring HABs. A further limitation is the persistent cloud cover, which in Chilean Patagonia can reach 50% and in northwest Patagonia is often higher [40]. The reduced availability of clear-sky observations hinders the generation of reliable matchup datasets, which are essential for validating remote sensing products. Nonetheless, given the large geographical extent and the isolation of certain areas of Patagonia, integrating the satellite-based monitoring of algal blooms with in situ and remote sensing information will enable better decision-making by end-users. This study showed that satellite images with enhanced spatial resolution can contribute to the design and implementation of HAB monitoring programs, especially in remote areas such as the Gulf of Penas in Chilean Patagonia. The development of remote sensing tools is critical in reducing the socio-economic and ecological impacts of HABs.

## 5. Conclusions

This study demonstrates the utility of integrating remote sensing and in situ measurements to characterize HABs in remote, data-scarce regions such as Chilean Patagonia. Despite its moderate cell density, the *Karenia* spp. bloom recorded in the Gulf of Penas during the austral summer of 2017 had severe ecological and economic impacts, highlighting the importance of the early detection of HABs. Sentinel-3 satellite imagery combined with oceanographic field data and classification algorithms such as the SVC can facilitate the identification and spatial mapping of bloom events in optically complex waters.

While persistent cloud cover and the geographical complexity of fjord systems pose challenges for the monitoring of HABs, our results support the incorporation of high-resolution satellite tools in early warning and monitoring programs. The successful implementation of satellite-derived indicators, such as FLH, NDCI, and RBD, along with the validation of machine learning approaches, opens up new avenues for improving HAB detection and risk management in southern Chile. Future work should focus on increasing the number and temporal resolution of in situ datasets to enhance algorithm performance and further reduce the rate of false positives/false negatives in bloom prediction models.

## Figures and Tables

**Table 1:** Coincidences between bloom observation and bloom prediction based on the computed RBD.

Station	Bloom	Computed RBD	Predicted Bloom	Coincidence
1	0	1.088	1	0
2	0	0.925	1	0
3	0	0.552	1	0
4	1	0.173	1	1
5	1	−0.698	0	0
6	0	0.015	0	1
7	1	1.328	1	1
8	0	0.691	1	0
9	1	0.742	1	1
10	0	0.515	1	0
11	0	−0.111	0	1
12	0	−1.306	0	1
			Total matches	6

**Table 2:** Coincidences between observed bloom events and blooms predicted using the *V* equation.

Station	Bloom	Computed *V*	Predicted Bloom	Coincidence
1	0	−1.000	0	1
2	0	−1.220	0	1
3	0	−1.001	0	1
4	1	1.001	1	1
5	1	−3.522	0	0
6	0	−2.029	0	1
7	1	0.079	1	1
8	0	−1.092	0	1
9	1	−2.160	0	0
10	0	−1.000	0	1
11	0	−0.777	0	1
12	0	−2.734	0	1
			Total matches	10

**Table 3:** Marginal PERMANOVA results based on the Jaccard dissimilarities of the properties (temperature, salinity, oxygen, and fluorescence) of the water column harboring the phytoplankton community (58 types), using 10,000 permutations for the hypothesis test.

Predictive Variables	DF	Sum of Squares	R^2^	Pseudo—F	*Pr* > *F*
Temperature	1	0.625	0.039	1.418	0.056
Salinity	1	0.638	0.040	1.448	0.049
Oxygen	1	0.617	0.038	1.401	0.069
Fluorescence	1	0.255	0.016	0.579	0.984
Residuals	31	13.54	0.862		
Total	35	15.84	1.000		

## Data Availability

The original contributions presented in this study are included in the article. Further inquiries can be directed to the corresponding author.

## Appendix A

### Spectral Band Characteristics

**Table A1:** Sentinel-3 Ocean and Land Colour Instrument (OLCI) band characteristics [82].

Band	λ Center (nm)	Width (nm)	Function
Oa01	400	15	Aerosol correction, improved water constituent retrieval
Oa02	412.5	10	Yellow substances, detrital pigments (turbidity)
Oa03	442.5	10	Chlorophyll absorption maximum, biogeochemistry, vegetation
Oa04	490	10	High chlorophyll
Oa05	510	10	Chlorophyll, sediment, turbidity, red tide
Oa06	560	10	Chlorophyll reference (chlorophyll minimum)
Oa07	620	10	Sediment loading
Oa08	665	10	Chlorophyll (2nd chlorophyll absorption maximum), sediment, yellow substances, vegetation
Oa09	673.75	7.5	Improved fluorescence retrieval and to account for smile together with the bands at 665 and 680 nm
Oa10	681.25	7.5	Chlorophyll fluorescence peak, red edge
Oa11	708.75	10	Chlorophyll fluorescence baseline, red edge transition
Oa12	753.75	7.5	O2 absorption, clouds, vegetation
Oa13	761.25	2.5	O2 absorption band, aerosol correction
Oa14	764.375	3.75	Atmospheric correction
Oa15	767.5	2.5	O2A, used for cloud top pressure, fluorescence over land
Oa16	778.75	15	Atmospheric/aerosol correction
Oa17	865	20	Atmospheric/aerosol correction, clouds, pixel co-registration
Oa18	885	10	Water vapor absorption reference band, common reference band with the SLSTR instrument, vegetation monitoring
Oa19	900	10	Water vapor absorption, vegetation monitoring (maximum reflectance)
Oa20	940	20	Water vapor absorption; atmospheric/aerosol correction
Oa21	1020	40	Atmospheric/aerosol correction

**Table A2:** Moderate Resolution Imaging Spectroradiometer (MODIS) [83,84].

Band	Wavelength (1)	Bandwidth (nm)	Function
8	415	405–420	Ocean color, phytoplankton, biogeochemistry
9	443	438–448	Ocean color, phytoplankton, biogeochemistry
10	490	483–493	Ocean color, phytoplankton, biogeochemistry
11	531	526–536	Ocean color, phytoplankton, biogeochemistry
12	565	546–556	Ocean color, phytoplankton, biogeochemistry
13	653	662–672	Ocean color, phytoplankton, biogeochemistry
14	681	673–683	Ocean color, phytoplankton, biogeochemistry
15	750	743–753	Ocean color, phytoplankton, biogeochemistry
16	865	862–877	Ocean color, phytoplankton, biogeochemistry

**Table A3:** Envisat MEdium Resolution Imaging Spectrometer (MERIS) band characteristics [85].

Band	Wavelength (1)	Bandwidth (nm)	Function
1	412.5	10	Yellow substances and detrital pigments
2	442.5	10	Chlorophyll absorption maximum
3	490	10	Chlorophyll and other pigments
4	510	10	Suspended sediment, red tides
5	560	10	Chlorophyll absorption minimum
6	620	10	Suspended sediment
7	665	10	Chlorophyll absorption and fluorescence reference
8	681.25	7.5	Chlorophyll fluorescence peak
9	708.75	10	Fluorescence reference, atmospheric corrections
10	753.75	7.5	Vegetation, cloud
11	760.625	3.75	Oxygen absorption R-branch
12	778.75	15	Atmosphere corrections

## Appendix B

### Algorithms

Chlorophyll *a* concentration

Morel et al. [86] developed the OC4Me algorithm to define the chlorophyll-*a* (Chl-*a*) concentration. This semi-analytical algorithm is based on the identification of the maximum band ratio (MBR) of bands 443 nm, 490 nm, and 510 nm vs. band 560 nm. The algorithm is the latest version of the MERIS pigment index algorithm described in Morel et al. [87]. The present study used Sentinel-3 bands Oa03 = 442.5 nm, Oa04 = 490 nm, and Oa05 = 510 nm, expressed as follows:

$$\log10\;(\mathrm{pigment}\;\mathrm{index})\;=\;\sum_{i=0}^{n}A_{i}{\left({log}_{10}Max\rho_{0}\right)}^{i}$$

where:

$$\mathrm{Max}\;\rho_{0}=\;\max\;[(\rho_{2,5}{)}_{0},\;(\rho_{3,5}{)}_{0},\;(\rho_{4,5}{)}_{0}]$$

$$\rho_{i,j}=R(\lambda i)/R(\lambda j)$$

λj is the green wavelength available with Sentinel-3 is λ6 = 560 nm.

λi is one of the three wavelengths in the blue and blue-green part of the spectrum, namely, λ3 = 442.5 nm, λ4 = 490 nm, λ5 = 510 nm (λ = band center wavelength).

Normalized Difference Chlorophyll Index (NDCI)

Mishra and Mishra [57] developed the NDCI to predict the Chl-*a* concentration from remote sensing data in estuarine and coastal waters. This index was calibrated and validated using MEdium Resolution Imaging Spectrometer (MERIS) datasets. The present study used Sentinel-3 band Oa08 = 665 and band Oa11 = 708.75. The NDCI was therefore expressed as follows:

$$NDCI=\frac{\left(Rrs708-Rrs665\right)}{\left(Rrs708+Rrs665\right)}$$

where Rrs708 is the remote sensing reflectance at 708 nm, and Rrs665 is the remote sensing reflectance at 665 nm.

Chlorophyll fluorescence

Letelier and Abbott [58] showed that the Moderate Resolution Imaging Spectroradiometer (MODIS) sensor specifications enable the observation of fluorescence at Chl-*a* concentrations as low as 0.5 mg m^−3^ at the sensor’s full resolution (nominally 1 km^2^ at nadir) under optimal viewing conditions. This is expressed as follows:

FLH = L_14_ − L _baseline_

where:

L_14_ = radiance in band 14

where, in MODIS:

L _baseline_ = L_15_ + (L_13_ – L_15_) * [(λ_13_ − λ_14_)/(λ_15_ − λ_13_)]

L = radiance

λ = band center wavelength

The central bands in MODIS are L13 = 653 nm; L14 = 681 nm; L15 = 750 nm.

The present study used Sentinel-3 band Oa08 = 665 nm, band Oa09 = 673.75 nm, and band Oa12 = 753.75 nm.

Diffuse Attenuation Coefficient (Kd)

The water column Kd for downwelling plane irradiance at 490 nm is an indicator of water transparency. The present study used the OK2-560 algorithm developed by Morel et al. [87], which is based on the 490–560 reflectance ratio, and the Sentinel-3 Ocean and Land Colour Imager (OLCI) bands Oa04 = 490 nm and Oa06 = 560 nm. This is expressed as follows:

$$\mathrm{Kd}\;(490)\;=\;K_{w}(490)\;+\;{10\;}^{\sum_{x=0}^{n}A_{x}{\left({log}_{10}\rho_{490,560}\right)}^{x}}$$

where:

*K_w_* (490) = 0.0166 m^−1^ and $\;\rho_{490,560}$ is the normalized quotient of water reflectance at 490 nm and 560 nm. The five *A_x_* coefficients have the following values: *A*_0_: −0.82789, *A*_1_: −1.64219, *A*_2_: 0.90261, *A*_3_: −1.62685, *A*_4_: 0.088504.

## Appendix C

### Appendix C.1. Phytoplankton Species Key

**Table A4:** Phytoplankton species included in the constrained analysis of proximities.

Scientific Name	Abbreviation
Dictyochophyceae	
*Vicicitus globosus*	Vglo
*Dictyocha speculum*	Dspe
Diatoms	
*Cerataulina pelagica*	Cpel
*Eucampia cornuta*	Ecor
*Chaetoceros criophilus*	Ccri
*Chaetoceros* spp.	Cspp
*Dactyliosolen fragilissimus*	Dfra
*Ditylum brightwellii*	Dbri
*Guinardia delicatula*	Gdel
*Guinardia striata*	Gstr
*Lauderia* spp.	Lspp
*Leptocylindrus danicus*	Ldan
*Odontella aurita*	Oaur
*Paralia sulcata*	Psul
*Rhizosolenia aff. setígera*	Rset
*Rhizosolenia styliformis*	Rsty
*Skeletonema* spp.	Sksp
*Thalassiosira subtilis*	Tsub
*Thalassiosira* spp.	Thsp
*Cylindrotheca closterium*	Cclo
*Grammatophora marina*	Gmar
*Navicula* spp.	Nasp
*Pennadas*	Penn
*Pseudo-nitzschia* cf. *australis*	Paus
*Pseudo-nitzschia* spp.	Pnsp
*Thalassionema frauenfeldii*	Tfra
Dinoflagellata (thecate)	
*Azadinium* spp.	Azsp
*Ceratium furca*	Cfur
*Ceratium fusus*	Cfus
*Ceratium lineatum*	Clin
*Dinophysis acuminata*	Dacu
*Dinophysis acuta*	Duta
*Dinophysis tripos*	Dtri
*Heterocapsa triquetra*	Htri
*Prorocentrum micans*	Pmic
*Protoceratium reticulatum*	Pret
*Protoperidinium brevipes*	Pbre
*Protoperidinium pellucidum*	Ppel
*Protoperidinium steinii*	Pste
*Protoperidinium* spp.	Pspp
*Pyrocistis* spp.	Pysp
*Scrippsiella* sp.	Scsp
*Torodinium teredo*	Tter
*Zygabicodinium lenticulatum*	Zyle
Dinoflagellata (athecate)	
*Cochlodinium* sp.	Cosp
Gymnodinials (unidentified)	Gyun
*Gyrodinium* sp.	Gysp
*Gyrodinium lachryma*	Gyla
*Gyrodinium spirale*	Gspi
*Karenia* cf. *mikimotoi*	Kmik
*Karenia* sp. 1	Ksp1
*Karenia* sp. 3	Ksp3
*Karenia* spp.	Kspp
*Karenia brevisulcata/digitata*	Kbre
*Kaenia papilionacea/brevis*	Kpap
*Warnovia* sp.	Wasp
*Pronoctiluca* sp.	Prsp
Others	
*Ciliates*	Cill
*Euglenophyta*	Eugl
*Nanoflagellates*	Nano
*Flagellate*	Flag
